# Screening chemical modulators of benzoic acid derivatives to improve lipid accumulation in *Schizochytrium limacinum* SR21 with metabolomics analysis

**DOI:** 10.1186/s13068-019-1552-2

**Published:** 2019-09-04

**Authors:** Zhipeng Li, Xueping Ling, Hao Zhou, Tong Meng, Jinjin Zeng, Wei Hang, Yanyan Shi, Ning He

**Affiliations:** 1 Department of Chemical and Biochemical Engineering, College of Chemistry and Chemical Engineering, Xiamen University, Xiamen, 361005 People’s Republic of China; 2 The Key Lab for Synthetic Biotechnology of Xiamen City, Xiamen University, Xiamen, 361005 People’s Republic of China; 3 Present Address: College of Food and Biological Engineering, Jimei University, Xiamen, 361021 People’s Republic of China

**Keywords:** *Schizochytrium*, Lipid, Benzoic acid derivatives, Metabolomics

## Abstract

**Background:**

*Schizochytrium* sp. is a marine fungus with great potential as an alternative commercial source of lipids rich in polyunsaturated fatty acids (PUFAs). To further increase lipid accumulation in *Schizochytrium* sp., the effect of exogenous additives has become one of the hotspots of current research. Although benzoic acid derivatives showed positive effects on lipid accumulation in *Schizochytrium*, the biochemical mechanism needs further investigation.

**Results:**

Four benzoic acid derivatives (sodium benzoate, *p*-aminobenzoic acid, *p*-methyl benzoic acid and folic acid) were screened and evaluated for their effect on lipid accumulation in *Schizochytrium limacinum* SR21. The lipid yield was increased by 56.84% with *p*-aminobenzoic acid (*p*-ABA) at a concentration of 200 mg/L among the four tested chemical modulators. The metabolomics analysis showed that 200 mg/L *p*-ABA was optimal for promoting glucose catabolism in glycolysis with an increase in the mevalonate pathway and a weakening of the tricarboxylic acid (TCA) cycle. Moreover, *p*-ABA increased NADPH generation by enhancing the pentose phosphate pathway (PPP), ultimately redirecting the metabolic flux to lipid synthesis. Fed-batch fermentation further proved that *p*-ABA could significantly increase the yield of lipid by 30.01%, reaching 99.67 g/L, and the lipid content was increased by 35.03%, reaching 71.12%. More importantly, the yields of docosahexaenoic acid (DHA) and eicosapentaenoic acid (EPA) were increased by 33.28% and 42.0%, respectively.

**Conclusion:**

The addition of *p*-ABA could promote the synthesis of tetrahydrofolate, enhancing NADPH, which ultimately promoted the flow of carbon flux to lipid synthesis. These findings provide a valuable strategy for improving the lipid accumulation in *Schizochytrium* by additives.

## Background

Polyunsaturated fatty acids (PUFAs), such as docosahexaenoic acid (DHA) and eicosapentaenoic acid (EPA), are a class of substances that are beneficial to the human body, could decrease the risk of some diseases, such as Alzheimer’s disease, inflammation and cardiovascular diseases, and have been widely used in the food and medicine fields [1–3]. The traditional source of DHA is fish oil. However, due to the sustainability of marine resources and environmental pollution (dioxins, and mercury) of fish, many researchers have focused on alternative sources of DHA. *Schizochytrium* sp., a marine fungus that has been an effective host for the sustainable generation of lipid-based bioproducts such as biodiesel [4–6], is also regarded as an effective fermentative microorganism for a new commercial source of DHA [7].

To further increase lipid accumulation and DHA yield in *Schizochytrium* sp., several strategies of fermentation process optimization have been performed. The regulation of lipids and polyunsaturated fatty acids by exogenous addition has become one of the hotspots of current research. The combination of 2 mg/L naphthoxyacetic acid and 20 mg/L jasmonic acid improved lipid accumulation up to 16.79% in *Schizochytrium* [8]. Yu et al. reported that 6-benzylaminopurine led to a 25.9% increase in lipid yield in *Schizochytrium* sp., reaching 52.8% of biomass [9]. In addition, 4 mg/mL gibberellin enhanced the biomass and lipid by 14.4% and 43.6% in *Aurantiochytrium* sp.YLH70, respectively [10]. However, the addition of such hormonal substances in the fermentation process of food-grade products might be controversial. In recent years, the effects of micronutrients, such as vitamins and trace elements, on lipid accumulation have been investigated. Ren et al. improved cell dry weight and DHA yield in *Schizochytrium* sp. HX-308by 16.16% and 30.44%, respectively, by adding 9 g/L ascorbic acid [11]. Wang et al. found that folic acid could promote the synthesis of lipids by *Mortierella alpine* by increasing NADPH [12]. The synthesis of PUFAs in *Schizochytrium* also requires the involvement of NADPH [13, 14]. *p*-Methyl benzoic acid promoted EPA production by suppressing methyl-directed desaturations from the Δ13 position in *Schizochytrium* sp [15]. Zhang et al. noted that the addition of *p*-methyl benzoic acid was able to increase the growth of oleaginous yeast, resulting in a higher lipid accumulation [16]. Sofia et al. reported that benzoic acid had a dramatic effect on intracellular phospholipid content, but the stress response mechanism was unclear [17].

In this study, four benzoic acid derivatives were chosen to explore their effects on the production of lipids by *Schizochytrium limacinum* SR21. Metabolomics analysis was adopted to investigate the biochemical mechanism.

## Results and discussion

### Effects of different benzoic acid derivatives on lipid yield

**Fig. 1:**
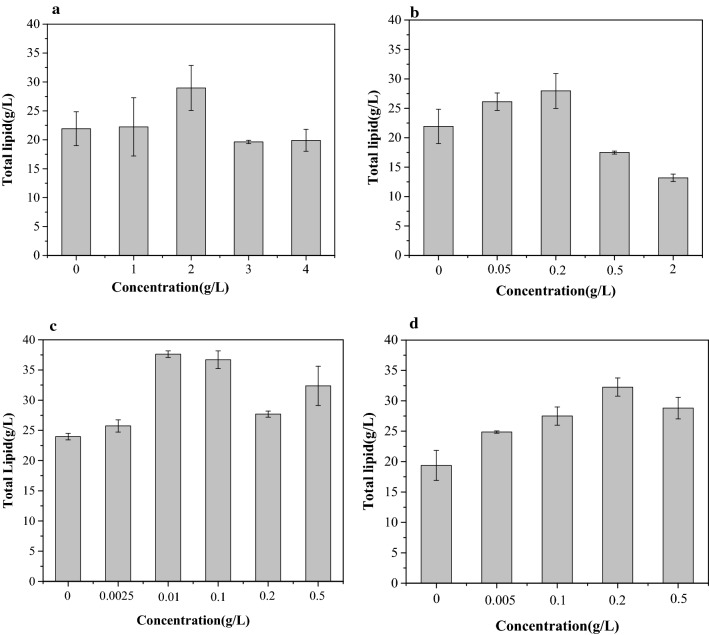
Total lipid accumulation of *Schizochytrium limacinum* SR21 supplemented with various concentrations of **a** SBA, **b** *p*-MBA, **c** FA, and **d** *p*-ABA

To screen the benzoic acid derivatives, *Schizochytrium limacinum* SR21 was cultured in fermentation medium supplemented with different concentration of the solution stocks. Consistent with Yue et al. study [18], no toxic effect of benzoic acid derivatives on growth was observed (Additional file 1: Fig. S1) if the addition of the chemical occurred at 48 h. As shown in Fig. 1, the lipid yield was influenced by different concentrations of benzoic acid derivatives. Notably, all of them, including sodium benzoate (SBA), *p*-aminobenzoic acid (*p*-ABA), *p*-methyl benzoic acid (*p*-MBA) and folic acid (FA), showed positive effects on lipid accumulation. The concentration of benzoic acid derivatives in the medium ranged from 0.0005 to 4 g/L. For the medium containing SBA, the lipid yield was increased by 32.12% in the 2 g/L SBA medium (Fig. 1a) compared with the control. The maximum lipid yield was obtained when 0.2 g/L *p*-MBA was added to the medium (Fig. 1b), and the addition of *p*-MAB caused a 32.15% increase in lipid yield. As shown in Fig. 1c, the highest lipid yield was observed when the medium contained FA at a concentration of 0.01 g/L, achieving an increase in the total lipid yield of 38.42%. More importantly, 0.2 g/L was the optimum concentration of *p*-ABA for lipid yield, causing a 56.84% increase in lipid yield. The results showed that *p*-ABA was the best benzoic acid derivative, and the optimal concentration was 0.2 g/L for the lipid yield.

There were differences in some structures of these four additives and in their function of promoting lipid accumulation. There was a certain competitive relationship between lipid accumulation and cell growth. When the growth of the cell is slow, it is in a period of rapid lipid accumulation [19]. Wang and Hirasaka noted that BA and *p*-MBA could inhibit the growth of microorganisms [15, 20] and then promote lipid synthesis and thereby change the proportion of lipids. On the other hand,* p*-ABA has similar functions to FA [16]. The main functional component of FA is *p*-ABA [21]. Chen et al. suggested that FA could promote lipid synthesis in microorganisms [12]. This may be the direct effect of *p*-ABA on lipid synthesis.

### Lipid profile analysis of enhanced total lipids

Finally, lipid profile analysis via GC was performed to evaluate whether the DHA content was different in the total lipid of *Schizochytrium* sp. after treatment with different concentrations of p-ABA (Additional file 1: Fig. S2). No significant changes in DHA content were observed in samples treated with different concentrations of *p*-ABA, suggesting that the productivity of DHA remained unchanged. Nevertheless, the total production of DHA could be increased due to the enhancement of lipid accumulation in *Schizochytrium* sp. after treatment with the chemical.

No significant changes in the lipid content in samples treated with different concentrations of *p*-ABA were found, suggesting that the productivity of PUFAs remained unchanged. Nevertheless, the total production of PUFAs could be increased due to the enhanced lipid accumulation in *Schizochytrium* sp. after treatment with the chemical. Similar related reports of microalgae and fungi have been published [23–27].

### Metabolite profiling of *Schizochytrium limacinum* SR21 with different concentrations of *p*-ABA treatment

The detected metabolites were mainly fatty acids, amino acids, organic acids, carbohydrates, alcohols, squalene, cholesterol, and so on, most of which were involved in the tricarboxylic acid (TCA) cycle, lipid synthesis and amino acid metabolism.

**Fig. 2:**
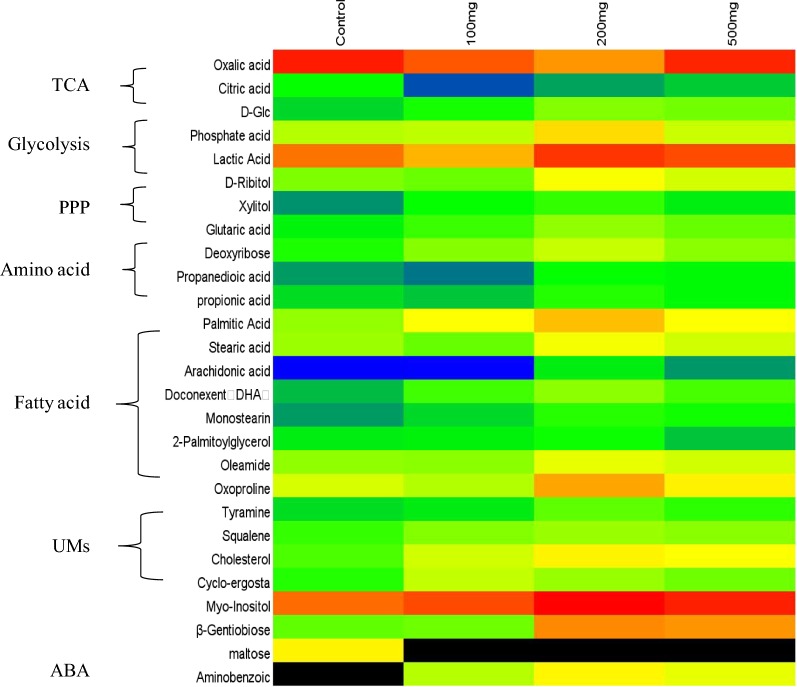
Comparative metabolite profile analysis of the fermentation process with and without *p*-ABA

As shown in Fig. 2, metabolite profiling of *Schizochytrium limacinum* SR21 was affected by different concentrations of *p*-ABA treatment. *p*-ABA was detected in the intracellular metabolites of the 100–500 mg/L *p*-ABA group, and its content was the highest in the 200 mg/L *p*-ABA group. This indicated that *Schizochytrium* sp. could absorb *p*-ABA into cells for metabolism, but there might be a threshold of *p*-ABA absorption (200 mg/L). On the other hand, the concentrations of glycolysis, the lactic acid synthesis pathway and other metabolites related to lipid synthesis with 200 mg/L*p*-ABA were the highest, which has the best effect on promoting the related metabolic pathways in lipid synthesis. Therefore, 200 mg/L *p*-ABA was selected for metabolomics analysis of the time series in the following experiments.

### Metabolite profiling of *Schizochytrium limacinum* SR21 with *p*-ABA treatment

**Fig. 3:**
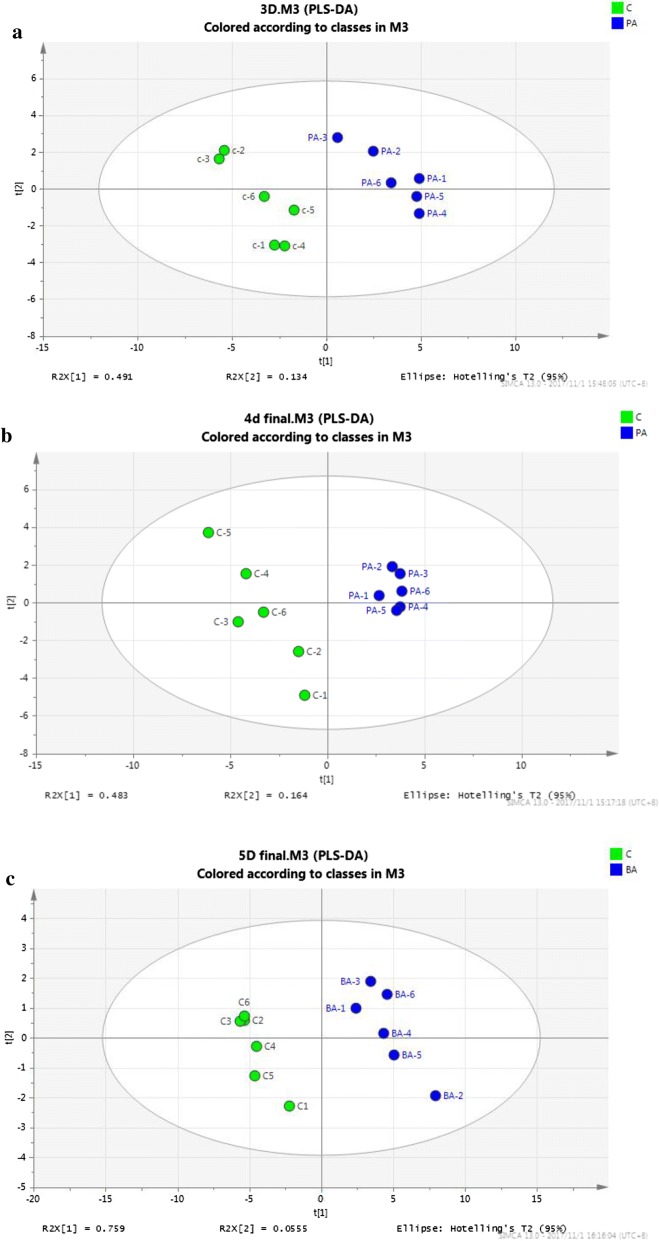
PLS-DA analysis of the *p*-ABA and control groups at **a** 72 h, **b** 96 h, and **c** 120 h

Through PLS-DA pairwise comparison, a significant separation of metabolic profiles between the control and *p*-ABA treatment (200 mg/L) groups at each time point was observed (Fig. 3a–c). The PLS-DA models were well constructed with excellent fit (Additional file 1: Fig. S3) and also built to determine the metabolic variability between two groups at each time point. As shown in VIP in Additional file 1: Fig. S3, 30 intracellular metabolites were selected from all mass spectra peaks, with VIP values > 1 and *P* values < 0.05, 28 of which could be identified as characteristic metabolites. By comparing PLS-DA at each time point, a significant separation of metabolite profiles was observed (Fig. 3a–c). This indicated that p-ABA could significantly affect the metabolic characteristics of *Schizochytrium*.

**Fig. 4:**
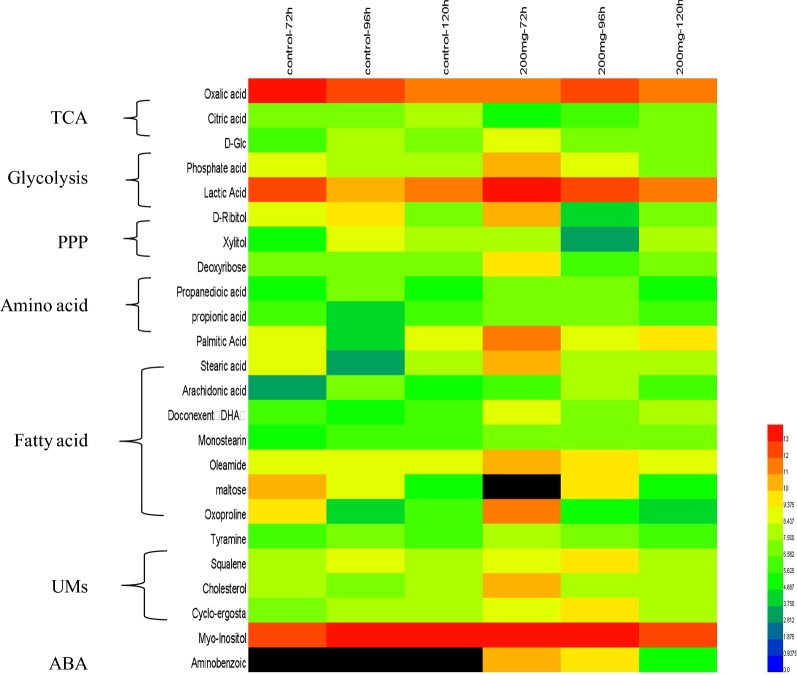
Heat map of the metabolite profile in the 200 mg/L *p*-ABA group at different time points

In this study, an analysis of the cluster heatmap was generated based on the relative content of metabolites (Fig. 4) to describe the effects of 200 mg/L *p*-ABA addition on the metabolites of *Schizochytrium*. The main metabolites were lipids, amino acids, organic acids, glycolytic substances, squalene and cholesterol. The *p*-ABA content in cells was the highest at 72 h in the group with* p*-ABA addition and then decreased gradually with time, which indicated that *p*-ABA was absorbed into cells and then metabolized and utilized gradually. No intracellular *p*-ABA was detected in the control group. Glucose and lactic acid during intracellular glycolysis decreased in both the control and additive groups at 72–120 h, which might be due to the depletion of extracellular substrates. The intracellular synthesis of lipids such as DHA, arachidonic acid (ARA) and saturated lipids also showed a downward trend, which was correlated with *p*-ABA substrate depletion.

**Fig. 5:**
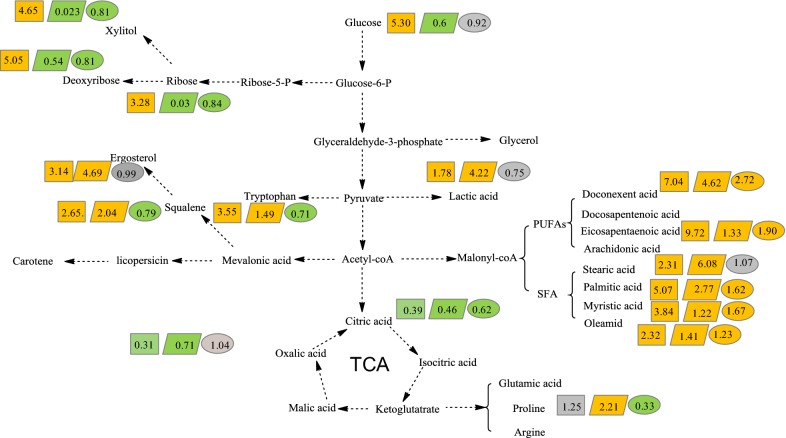
Changes in metabolites between the *p*-ABA group and the control group. Yellow: normalized contents of metabolites compared to those in the control group: increase (*P* < 0.05), green; decrease (*P* < 0.05), gray; no significant change (*P* > 0.05); rectangle: 72 h, parallelogram: 96 h, oval: 120 h

To analyze the effects of *p*-ABA on the metabolic process of *Schizochytrium*, the differential metabolites were mapped into the metabolic pathway, as shown in Fig. 5.For the *p*-ABA additive group, the intracellular glucose content in the glycolysis pathway was 5.3-fold higher than that in the control group at 72 h and 0.6-fold lower at 96 h. This suggests that the addition of *p*-ABA accelerated the absorption of glucose and that the absorbed glucose was rapidly consumed in the subsequent metabolic process. This consumption may provide more substrates for downstream metabolic pathways, such as the mevalonate pathway, lipid synthesis pathway and pentose phosphate pathway (PPP). Therefore, squalene and ergosterol in the mevalonate pathway increased 2.65- to 3.14-fold in the *p*-ABA additive group. Regarding intracellular lipid synthesis, both saturated fatty acids (SFAs) and PUFAs increased by 1.22- to 9.72-fold in the *p*-ABA additive group, which was consistent with the increase in total lipid in Fig. 1.

One of the main functions of folic acid is to promote the utilization of metabolites in the PPP, such as ribose and xylose, to produce DNA and RNA [28]. Figure 5 shows that the addition of *p*-ABA increased ribose 3.28-fold and deoxyribose 5.05-fold at 72 h, which then decreased to 0.03- and 0.023-fold at 96 h, respectively. This indicated that *p*-ABA promoted the metabolic flux through PPP at the initial stage of addition, but over time, *p*-ABA was used to synthesize folic acid and accelerated the utilization of these metabolites. Additional file 1: Fig. S4 shows the gene expression related to folic acid synthesis. The results suggested that the addition of *p*-ABA significantly increased the expression of the folate synthase gene (dihydrofolate reductase, Additional file 1: Fig. S4A) at 96 h and the tetrahydrofolate dehydrogenase gene (Additional file 1: Fig. S4B). The increase in the two enzymes accelerated the metabolism of the PPP, and the enhancement of tetrahydrofolate dehydrogenase and the PPP in the process was also observed, and more NADPH substrates could be provided [12]. In general, NADPH is the key substrate for lipid synthesis. Some studies have noted that NADPH from the PPP can increase the contents of PUFAs from the polyketide synthase (PKS) pathway [14]. Chen et al. also showed that the addition of NADPH could promote lipid production [29]. For this study, *p*-ABA was able to promote the PPP and folate synthase activity and then increase the NADPH supply, supporting the ability of the PKS and fatty acid synthase (FAS) pathways to synthesize lipids. This may be the reason for the increase in lipid in Fig. 1.

In addition, Wagner’s study has shown that folic acid is an important donor of carbon units [30]. At the beginning of lipid synthesis, one carbon unit is required from acetyl coenzyme A to malonyl coenzyme A. There is no direct evidence that folic acid can provide the one carbon unit for this purpose in *Schizochytrium*. However, the increased activity of folic acid-related enzymes indirectly proved that folic acid may be an important factor in lipid synthesis.

### Fed-batch fermentation of *Schizochytrium limacinum* SR21 with ABA treatment

**Fig. 6:**
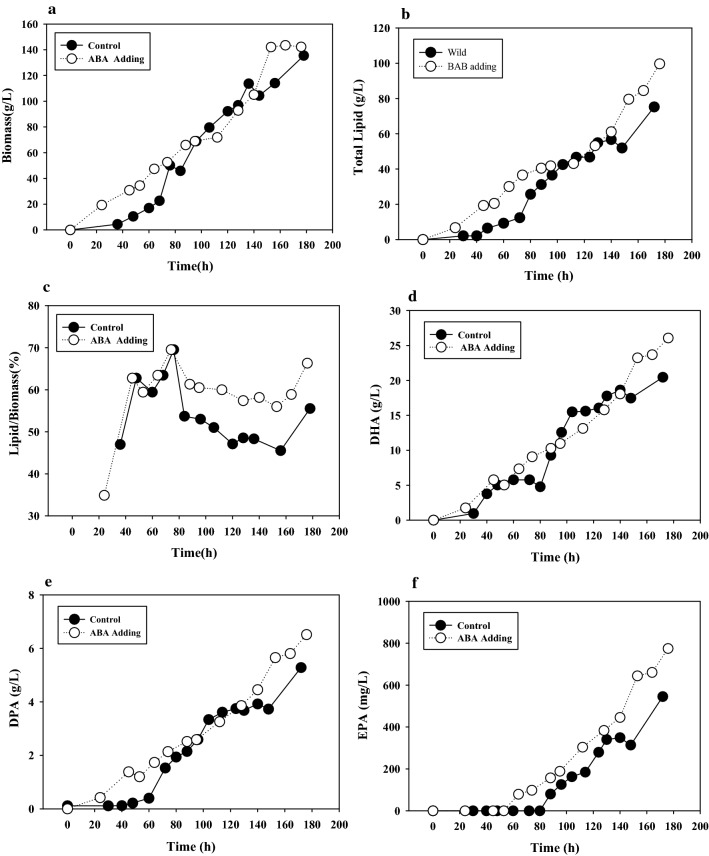
Results from the fed-batch fermentation of the *p*-ABA and control groups for **a** biomass, **b** total lipid, **c** lipid proportions, **d** DHA, **e** DPA and **f** EPA yield

To validate the effect of *p*-ABA on the lipid content and improve the lipid yield, 200 mg/L *p*-ABA was added to a fed-batch fermentation at 96 h. The biomass showed no significant difference (Fig. 6a),while the lipid yield was increased by 30.01% in the *p*-ABA addition group, reaching 99.67 g/L (Figure 6b). Fig. 6c further shows that the proportion of lipids in the biomass reached 71.12% and increased by 35.03%. These results indicated that *p*-ABA promoted both lipid yield and lipid proportion in *Schizochytrium* SR21, which were much higher than those reported by Wang et al. [8] and Yu et al. [9, 10]. Moreover, the yields of DHA and EPA were increased by 33.28% and 42.0%, respectively (Fig. 6d, f). This further illustrated the positive effect of p-ABA on PUFA production in *Schizochytrium*.

## Conclusions

The addition of four benzoic acid derivatives (sodium benzoate, *p*-aminobenzoic acid, *p*-methyl benzoic acid and folic acid) promoted the production of lipids in *Schizochytrium* *limacinum* SR 21. Among them, *p*-aminobenzoic acid increased the lipid yield by 56.84%. Metabolomics analysis showed that the biochemical mechanism related to glycolysis, the pentose phosphate pathway and lipid synthetic pathways (PKS and FAS). Fed-batch fermentation in a 3.6-L fermenter further proved that *p*-ABA could significantly increase both lipid yield by 30.01% and lipid content by 35.03%. Meanwhile, the yields of DHA and EPA were enhanced as well.

## Methods

### Strain, culture and benzoic acid derivative treatment

*Schizochytrium limacinum* SR21 was obtained from the American Type Culture Collection (ATCC, USA). This strain was maintained on seed broth agar plates. The fermentation and seed broth were the same as those used in our previous study [31]. After three generations of cultivation, the seed culture (4%, v/v) was then transferred to fermentation broth and incubated at 28 °C, 200 rpm for 120 h or more.

All benzoic acid derivatives used in this study were of analytical purity. Sodium benzoate (SBA), *p*-aminobenzoic acid (*p*-ABA), *p*-methylbenzoic acid (*p*-MBA) and folic acid (FA) were purchased from Sigma-Aldrich (St. Louis, USA). All reagents were stored at 4 °C until use. SBA, p-ABA, MBA, and FA were dissolved in ethanol. All solutions were sterilized using 0.22-μm polyvinylidene fluoride or polyethersulfone syringe filters (Millipore, MA, USA).

All chemical additive stock solutions were added to the medium at 48 h of fermentation cultivation. The concentrations of each chemical additive, including SBA, *p*-ABA, *p*- MBA, and FA, were 0.0025 g/L–2 g/L, as shown in Table 1. The data were analyzed by the means and standard deviations of triplicates. Each treatment was repeated three times to confirm the results.

**Table 1:** The concentration gradient with the addition of benzoic acid derivatives (final concentration)

Additive	SBA	*p*-MBA	FA	*p*-ABA
Concentration (g/L)	0	0	0	0
	1	0.05	0.0025	0.05
	2	0.2	0.01	0.1
	3	0.5	0.1	0.2
	4	2	0.5	0.5

### Lipid extraction and fatty acid composition analysis

A total of 3 mL of fermentation broth was mixed with 4 mL of HCl (12 N) and incubated in a water bath at 65 °C for 50 min. Total lipids from the mixture were extracted four times with 4 mL of *n*-hexane, and then the collected extracts were mixed and purified by the nitrogen blowing method at room temperature [32].

Fatty acid methyl esters (FAMEs) were prepared according to the methods of Kueiling et al. and Ren et al. [33, 34] with modifications: heptadecanoic acid methyl (16 g/L, Sigma, USA) as an internal standard was added to the tubes and mixed by vortexing for 1 min. The upper phase containing FAMEs was applied to a gas chromatograph (Agilent GC 7890, USA) equipped with a 100 m × 0.25 mm capillary column (SP™-2560, USA). Peaks were identified with authentic standards of fatty acid methyl esters (Sigma, USA).

### Quantitative real-time PCR (qRT-PCR) analysis

Total RNA was extracted by ZR Fungal/Bacterial RNA MicroPrep (ZYMO, USA) from 2 mL of *Schizochytrium* sp. cells at 72 h, 96 h, and 120 h. The cDNA was prepared with an EasyScript First-Strand cDNA Synthesis SuperMix (Transgen Biotech, China) according to the manufacturer’s protocol. qPCR was performed using a TransStart Top Green QPCR SuperMix (Transgen Biotech, China). The obtained cDNA was diluted to 100 ng/mL for further real-time PCR analysis. The qRT-PCR reaction was performed in a 20-μL mixture using a Transtart Top Green qPCR Supermix Kit (TransGen Biotech, China). A StepOne Real-time PCR System (Applied Biosystems) was utilized to detect the differential expression levels of genes [35]. The primers used for qRT-PCR are listed in Additional file 1: Table S1. Reactions without the template were used as negative controls.

### Preparation of metabolomic samples

Five milliliters of cells was sampled during batch fermentation at 72 h, 96 h, and 120 h. The collected cells were quenched, and cellular contents were extracted according to our previous study with moderate modifications. The sample was quickly mixed with 60% cold methanol (− 40 °C, v/v) to capture the metabolism of cells instantaneously and then centrifuged at 5000×*g* and − 4 °C for 5 min. Next, the cell pellet was washed twice with cold physiological saline and then stored at − 80 °C until use [35].

Samples were ground to a fine powder under liquid nitrogen. Then, 0.5 mL of prechilled methanol (− 40 °C) was added to 0.1 g of cell powder and mixed thoroughly for 30 s. The mixture was centrifuged at 8000×*g* and − 4 °C for 5 min, and the supernatant was collected. An additional 0.5 mL of prechilled methanol (− 40 °C) was added to the cell pellet, and the cell pellet was shaken vigorously for 30 s, followed by centrifugation at 8000×*g* and 4 °C for 5 min. Both supernatants were pooled together and dried in a vacuum freeze dryer and stored at − 80 °C for further detection.

### Derivatization and GC–MS analysis

To investigate the mechanism by which benzoic acid derivatives enhance the lipid yield of *Schizochytrium limacinum* SR21, a GC–MS method combined with multivariate analyses was used to indicate the changes in metabolites in the presence of p-ABA at different concentrations. Before GC–MS analysis, sample derivatization was performed according to the method of our previous study [36], with moderate modifications. The samples obtained as described above and 40 μL of internal standard (methyl heptadecanoic acid in *n*-hexane, 1 mg/mL) were mixed and dried in a vacuum freeze dryer. Methoxyamine hydrochloride (50 μL) in pyridine (20 mg/mL) was added to the dried sample and incubated at 37 °C for 2 h. Next, the sample was silylated for 2 h at 37 °C by adding 50 μL of *N*-methyl-*N*-(trimethylsilyl) trifluoroacetamide (MSTFA, Sigma, Burlington, USA) and vortex mixing for 30 s.

A 1-μL sample was used with splitless injection into the Agilent 7890–5975C GC–MS equipped with a fused silica capillary column (30 m × 0.25 mm × 0.25 μm, DB-5MS, Agilent). The column and ion source temperatures were set according to our previous study. The mass scan range was 50–600 *m/z*.

### Compound identification and data processing

The peak area value of each metabolite was calculated and normalized based on the internal standard peak intensity and concentration. The intracellular metabolite level was calculated as the ratio of the metabolite concentration to the corresponding biomass. The peak with a reverse similarity index (RSI) value ≥ 700 (on a scale from 1 to 999) was identified as a metabolite (detailed information is shown in Additional file 1: Table S2). Some different authentic standards (glutamic acid, citric acid, glucose, squalene, myo-insitol, EPA, DHA and sterol) were analyzed separately under the same conditions as above by GC–MS to validate the major metabolite annotation results. The dataset was normalized using division of the intensity of the peaks in each sample by the intensity of the internal standard peak and then by the quantity of internal standard on the same chromatograph [37–40].

The levels of all identified metabolites in different sampling protocols were clustered and are shown in the heat map using Cluster software (HemI 1.0, Chinese Academy of Sciences, China). A supervised partial least squares discriminant analysis (PLS-DA) was subsequently performed to identify the metabolites contributing to differences between the control and treatment. A metabolite with a variable influence on the projection value (VIP) higher than 1 indicates a significant contribution to the separation of groups in the PLS-DA models. One-way analysis of variance (ANOVA) tests were performed to analyze the distribution of normalized contents of metabolites in this study.

### Fed-batch fermentation

Fed-batch fermentation of *p*-ABA supplementation and control was carried out in a 3.6-L fermentor. The initial culture broth was the same as that used in our previous study [36]. The seed culture (10%, v/v) was then transferred to the fermentor with a working volume of 1.8 L. When the glucose concentration was below 20 g/L, additional glucose was supplemented to 60 g/L. Considering that artificial seawater (AS) would be diluted by feeding glucose, 20% AS (v/v) and *p*-aminobenzoic acid could be added on the 4th day. The other fermentation conditions were the same as those in the study by Li et al. [36].

## Supplementary information

**Additional file 1.** Additional figures and table.

## Data Availability

Not applicable.

## Abbreviations

PUFAs: polyunsaturated fatty acids
SFAs: saturated fatty acids
DHA: docosahexenoic acid
EPA: eicosapentaenoic acid
PPP: pentose phosphate pathway
PKS: polyketide synthase
FAS: fatty acid synthase
*p*-ABA: *p*-aminobenzoic acid
TCA: tricarboxylic acid
SBA: sodium benzoate
FA: folic acid
*p*-MBA: *p*-methylbenzoic acid
FAMEs: fatty acid methyl esters
RSI: reverse similarity index
ANOVA: one-way analysis of variance
PLS-DA: partial least squares discriminant analysis
ATCC: american type culture collection
