# Supplementary material for: Screening chemical modulators of benzoic acid derivatives to improve lipid accumulation in Schizochytrium limacinum SR21 with metabolomics analysis
Source: Biotechnol Biofuels. 2019 Sep 4;12:209. doi: 10.1186/s13068-019-1552-2 (PMC6724347; doi:10.1186/s13068-019-1552-2)
Supplement: Supplementary file 1 — Additional file 1. Additional figures and table. [file 13068_2019_1552_MOESM1_ESM.docx]

**Fig. S1 Biomass of *Schizochytrium* *limacinum* SR21 supplemented with various concentrations (a) BA (b) MBA (c) FA (d) ABA**

**

**Fig. S2 The lipid profiles in *Schizochytrium* *limacinum* SR21 under different concentrations of ABA**

**Fig. S3 The VIP value of specific metabolites.(A)-(C): 72nd -120th h**

**Fig.S4 Quantitative RT-PCR of the gene expression (A) dihydrofolate reductase (DHFR) gene and (B) tetrahydrofolate dehydrogenase (MTHFD)**

| **Table S1 RT-PCR primers used in this study** | | |
| --- | --- | --- |
| Gene | Primer name | Primer sequence(5'-3') |
| DHFR | DH-F | AACACCCAGCTACTGCCATACC |
|  | DH-R | CCTCCACTGCCAATCTTTG |
| MTHFD1 | MT1-F | CGAGAAGGGCTACCAGGTTC |
|  | MT1-R | GAGACCACAGGTACGGATGG |
| MTHFD2 | MT2-F | AGCCTCGTCGTAGCCCATCT |
|  | MT2-R | TGCAGCCAGCTCCTCAAGAT |
